# Foreword

**DOI:** 10.1080/15438600490447816

**Published:** 2004


*Experimental Diab. Res.*, 5:3–5, 2004
Copyright © Taylor and Francis Inc.
ISSN: 1543-8600 print / 1543-8619 online
DOI: 10.1080/15438600490447816

Taylor & Francis
Taylor & Francis Group

### Foreword

"Nature abhors a vacuum," but does it always assign a function to all peptides produced in the body? When I started my career in Neuroendocrinology I learned that there were many physiologic functions for which no peptide or hormone had been found, many peptides for which no physiologic function had been established, that certain peptides simply served as markers but had no function and others had established functions. Yet, as techniques evolved to probe in greater depth into the mysteries of the human body new functions were being discovered. It seems now that C-peptide may fulfill this last category.

The connecting peptide or C-Peptide was first described by Steiner (1967) in Chicago as a byproduct of insulin biosynthesis. The C-peptide links the insulin A and B chains in proinsulin, providing thereby a means to promote their efficient folding and assembly in the endoplasmic reticulum during insulin biosynthesis. It further facilitates the intracellular transport, sorting and proteolytic processing of proinsulin into biologically active insulin in the mature secretory granules of the B cells of the islets of Langerhans. C-peptide functions to allow proper alignment of the A and B chains of insulin and is stored in the soluble compartment of the secretory granules in association with chromogranin and IAPP and a number of the proinsulin/insulin intermediates such as those cleaved by the prohormone convertases PC2 and PC1/3. They in turn cleave insulin from the B and A chains of insulin respectively. C-peptide in mammalians is about 31 aminoacids long and has a central glycine-rich region, which allows it to fold like a concertina, permitting the appropriate alignment of the A and B chains for insulin to form its disulfide bonds and achieve its tertiary structure. Remarkably, the evolutionary related IGFs also contain a C-peptide, which is shorter in length, but C-peptide in proinsulin has a unique conservatism of structure testifying to its important role in peptide biosynthesis. C-peptide facilitates its own excision from proinsulin during maturation to insulin without which there would be reduced biological activity of insulin. Failure of this processing is seen as a defect in type two diabetes and the extreme example occurs in insulin secreting tumors in which the Proinsulin/insulin ratio is markedly increased.

For years no biological function was ascribed to C-peptide, instead the differences in its half-life and its distinction from beef and porcine C-peptide allowed assays to be developed which could distinguish epitopes present on the different C-peptides which allowed measurements even in the presence of these administered insulins and antibodies The differences in insulin half life of about 3 minutes vs. that of C-peptide of around 30 minutes also allowed for single measurements to be made as a means of reflecting upon $\beta$ cell mass and quantitated insulin secretory rates in vivo as a means of distinction between type 1 and 2 diabetes. Autonomous insulin overproduction in tumors was evaluated by means of C-peptide suppression tests; latterly, stimulation of C-peptide using meals, arginine or glucagon has been introduced into the clinical arena in an attempt to quantitate $\beta$ cell mass.

The high degree of conservation and overall length of C-peptide might argue in favor of a biological activity outside of insulin processing. This region of proinsulin accepts mutations at a rate 12–15 times greater than the evolution of both insulin and the IGFs suggesting that the molecule is not constrained by a need to interact with specific target tissues or membrane receptors. It has been accepted for decades that for any hormone to exert specific cellular effects a specific receptor should be identifiable at the cell surface and that it binds in a saturable, reversible manner and activates specific cellular events. The biologic actions found with C-peptide were first explained by non-chiral interactions with other molecules at the membrane surface such as insulin for which there appeared to be a mandatory need (Ido et al., 1997). The search for a specific receptor remained elusive until Rigler et al. (1999) and Henriksson et al. (2001) using fluorescence correlation spectroscopy sensitive enough to detect binding were able to demonstrate "receptors" on cultured renal tubular cells and saphenous vein endothelial cells. The binding was inhibited by pertussis toxin suggesting that binding was in some way coupled to G protein activaton. Thus, it was suggested that C-peptide signaling included activation of a specific pertussis toxin-sensitive G protein coupled receptor activation of calcium dependent intracellular signaling (Wahren and Jörnvall, 2003). The binding of C-peptide has an association constant of around $3.10^9$ $M^{-1}$ and saturation occurs at 1nM suggesting that a biological role would not be seen in normal subjects but only those deficient in C-peptide. It is still, however, not clear whether the actions of C-peptide are due to the receptor binding or the chiral interactions with other ligands. C-peptide stimulates glucose transport in human skeletal muscle (Zierath et al., 1996), increases glycogen synthesis and aminoacid uptake in L6 myoblasts (Grunberger et al., 2001), enhances $NA^+$,$K^+$-ATPase activity in rat sciatic nerve, granulation tissue, red blood cells, pancreatic islets, renal cells and other tissues. More importantly it activates endothelial nitric oxide synthase releasing NO from bovine aortic endothelial cells (Wahren et al., 2000). Nonetheless, it is clear that C-peptide alone or in combination with a permissive amount of insulin mimics several of insulin's effects. In insulin sensitive myocytes (L6 myoblasts), differentiated myocytes, adipocytes, neuroblastoma cells the signal pathways of C-peptide are similar to those of insulin-including the predominantly metabolic pathways involving PI-3 kinase and the growth promoting pathways involving MAP kinases. The receptor through which the C-peptide activities are transmitted remains elusive and will be an important program for future elucidation if C-peptide is to enter a biologic market. Nonetheless a number of physiological functions include stimulation of $NA^+/K^+$ ATPase activity, altered neural and vascular function (Wahren et al., 1994; Ido et al., 1997; Kitamura et al., 2001). While it still seems unlikely that C-peptide will bind to lipid rich plasma membranes there are a number of other means whereby it could modulate cellular responses and thereby participate in metabolic events, or growth promoting or anti-apoptotic events important in the biological stability of man.

There has been fairly uniform agreement that there is a reduction in $Na^+/K^+$ ATPase activity in red blood cells and other tissues in diabetes. Vague and colleagues argue cogently that this is due to the deficiency of C-peptide in type 1 diabetes, that it is corrected by C-peptide administration and is a direct effect of C-peptide in vitro. This, of course would have important ramifications on endothelial function and red blood cell deformability which affect tissue oxygenation and is critical to the viability of many tissues in people with diabetes.

There is a profound reduction in neurovascular perfusion of peripheral tissues including nutritive flow to skin in diabetes (Vinik et al., 2001). While this may be a consequence of prolonged hyperglycemia, increasing evidence supports the notion that disturbed blood flow precedes the development of hyperglycemia, occurs with impaired glucose tolerance (IGT) and even in the presence of the dysmetabolic syndrome in which resistance to the action of insulin is present and cosegregates with the disordered microvascular function. Fort and Kunt in this issue highlight the role of C-peptide in microvascular function and demonstrate that it may be feasible to correct the conduit (brachial artery, resistance (forearm) and nutritive (finger) deficits in blood flow in diabetes with C-peptide. It will, of course, require carefully designed structures with appropriate endpoints to validate these observations and clearly it will be necessary to define basal C-peptide status if the outcomes are to prove successful and not hamstrung by lack of appreciation of this relationship.

There may, however, be additional effects of C-peptide on microvascular integrity. Increased extracellular matrix protein deposition and capillary basement membrane thickening are characteristic features of diabetic retinopathy. It seems that oncofetal fibronectin synthesis is increased in retinas of a model of type 1 diabetes the BB/Wor rat secondary to an increase in TGF-$\beta$ and endothelin-1. C peptide abrogates the increase in oncofetal FN without changing the TGF-$\beta$ or endothelin transcripts suggesting a very distal role in ameliorating retinopathy. It was, however, without effect in the BB-ZDR-Wor rat retina model of type 2 diabetes again emphasizing an action only in depleted C-peptide models and dictating specificity in future study designs.

It has recently been established that the pathogenesis of diabetic neuropathies is complex and that no single mechanism can be held responsible for the manifestations of the diverse forms of the condition (Cameron et al., 2001; Sima et al., 2003; Vinik et al., 2004). Moreover, clinical evidence has supported the notion that C-peptide deficiency contributes to the severity thereof. Animal studies using the BB-Wor as a model of type 1 diabetes and the BB-ZDR-Wor as a model of type 2 diabetes have further elucidated differences in the pathogenesis of these conditions (Sima and Shafrir, 2003). As would be expected for the effects of C-peptide on $Na^+/K^+$ ATPase, endothelial NO and endoneurial blood flow, C-peptide significantly prevents both motor and sensory nerve conduction velocity deterioration as well as decreasing thermal hyperalgesia, a function of small unmyelinated C fibers (Sima et al., 2001; Sima, 2003). These effects are only partial and in the type 1 model appear to improvde the axonal degeneration and paranodal degenerative changes. In a small study in humans Ekberg et al. (2003) demonstrated that replacement with C peptide (600 nmol/day) resulted in significant improvement in nerve function with improved sensory nerve conduction velocities, and vibratory perception threshold within 3 months compared with insulin alone. There is also an improvement in autonomic function and warm thermal perception thresholds. Again this is due to the "insulinomimetic" action of C-peptide and does not occur with insulin alone. These effects may be mediated by enhancement of endoneurial blood flow by a NO and not a prostaglandin-mediated mechanism but appear to be independent of changes in oxidative stress. The changes in small fiber function such as thermal hyperalgesia apparently being mediated directly by NO are likely to be due to direct effects of C-peptide on neurotophism involving NGF, its receptors, IGFs, substance P, calcitonin gene related protein or as yet other non-identified peptide mediators of pain. The ability of C-peptide to restore damaged nerve function suggests that it may effectively induce nerve regeneration. In addition a variety of genes and their protein products have been implicated including the early response genes such as a c-fos, cytoskeletal proteins e.g. laminin, various cytokines e.g. IL-6, restoration of microfilament downregulation and upregulation of tubulin genes. At the paranode, proteins such as caspr (which interacts with spectrin, actin and contactin) are regulated through the activity of PI-3 kinases which are down-regulated in the type 1 diabetes model and correctible with C-peptide. (Sima et al., 2004). It also appears to have salutary effects on neuronal apoptosis thereby providing a rationale for its possible role in the protection against massive cerebral infarction with strokes (Li and Sima, 2004). In models of type 2 diabetes, which seem to exhibit a reduction in endoneurial blood flow and evidence of oxidative stress, C-peptide appears of fail its ability to alter nerve function nor is there a clear relationship between the altered endoneurial blood flow and nerve function.

Thus a skeletal peptide that once achieved fame and notoriety with the discovery of a pivotal role in regulating hormone secretion may be coming of age and entering the neuroendocrine stratosphere as a useful marker of $\beta$ cell integrity, a useful measure to distinguish type 1 from type 2 diabetes as well as the diagnosis of insulinomas. The provocative data presented in the accompanying manuscripts are sufficient to tantalize even the most discerning critical minds into wondering if the deity in his or her wisdom once again indulged in conservation and utilized one peptide structure to serve us in more than one way.

### REFERENCES

Cameron, N. E., Eaton, S. E. M., Cotter, M. A., and Tesfaye, S. (2001) Vascular factors and metabolic interactions in the pathogenesis of diabetic neuropathy. *Diabetologia,* **44,** 1973–1988.

Ekberg, K., Brismar, T., Johansson, B. L., Jonsson, B., Lindstrom, P., and Wahren, J. (2003) Amelioration of sensory nerve dysfunction by C-Peptide in patients with type 1 diabetes. *Diabetes,* **52,** 536–541.

Grunberger, G., Qiang, X., Li, Z., Mathews, S. T., Sbrissa, D., Shisheva, A., and Sima, A. A. F. (2001) Molecular basis for the insulinomimetic effects of C-peptide. *Diabetologia,* **44,** 1247–1257.

Henriksson, M., Pramanik, A., Shafqat, J., Zhong, Z., Tally, M., Ekberg, K., Wahren, I., Rigler, R., Johansson, J., and Jörnvall, H. (2001) Specific binding of proinsulin C-peptide to intact and to detergent-solubilized human skin fibroblasts. *Biochem. Biophys. Res. Commun.,* **280,** 423–427.

Ido, Y., Vindigni, A., Chang, K., Stramm, L., Chance, R., Heath, W. E., DiMarchi, R. D., Di Cera, E., and Williamson, J. R. (1997) Prevention of vascular and neural dysfunction in diabetic rats by C-peptide. *Science,* **277,** 563–566.

Kitamura, T., Kimura, K., Jung, B. D., Makondo, K., Okamoto, S., Canas, X., Sakane, N., Yoshida, T., and Saito, M. (2001) Proinsulin C-peptide rapidly stimulates mitogen-activated protein kinases in Swiss 3T3 fibroblasts: requirement of protein kinase C, phosphoinositide 3-kinase and pertussis toxin-sensitive G-protein. *Biochem. J.,* **355,** 123–129.

Li, Z.-G., and Sima, A. A. F. (2004) C-peptide and central nervous system complications in diabetes. *Exp. Diab. Res.,* **5,** 79–90.

Rigler, R., Pramanik, A., Jonasson, P., Kratz, G., Jansson, O. T., Nygren, P., Stahl, S., Ekberg, K., Johansson, B., Uhlen, S., Uhlen, M., Jörnvall, H., and Wahren, J. (1999) Specific binding of proinsulin C-peptide to human cell membranes. *Proc. Natl. Acad. Sci. U.S.A.,* **96,** 13318–13323.

Sima, A. A. F., Zhang, W., Sugimoto, K., Henry, D., Li, Z., Wahren, J., and Grunberger, G. (2001) C-peptide prevents and improves chronic Type I diabetic polyneuropathy in the BB/Wor rat. *Diabetologia,* **44,** 889–897.

Sima, A. A. F. (2003) C-peptide and diabetic neuropathy. *Expert Opin. Investig. Drugs,* **12,** 1471–1488.

Sima, A. A. F., Li, Z.-G., and Zhang, W. (2003) The insulin-like growth factor system and neurological complications in diabetes. *Exp. Diabesity. Res.,* **4,** 235–256.

Sima, A. A. F., and Shafrir, E. (2003) Editorial. *Exp. Diabesity. Res.,* **4,** i.

Sima A. A. F., Zhang, W., and Grunberger, G. (2004) Type 1 diabetic neuropathy and C-peptide. *Exp. Diab. Res.,* **5,** 65–77.

Steiner, D. F. (1967) Evidence for a precursor in the biosynthesis of insulin. *Trans. N. Y. Acad. Sci.,* **30,** 60–68.

Vinik, A., Erbas, T., Park, T., Stansberry, K., Scanelli, J., and Pittenger, G. (2001) Dermal neurovascular dysfunction in type 2 diabetes. *Diabetes Care,* **24**(8), 1468–1475.

Vinik, A., Pittenger, G., Barlow, P., and Mehrabyan, A. (2004) *Diabetic neuropathies an overview of clinical aspects, pathogenesis, and treatment*. LeRoith, D., Taylor, S., and Olefsky, J. Diabeties Mellitus (3rd Edition), 1331–1363. Philadelphia: Lippincott Williams and Wilkins.

Wahren, J., Johansson, B. I., and Wallberg-Henriksson, H. (1994) Does C-peptide have a physiological role? *Diabetologia,* **37**(Suppl 2), S99–107.

Wahren, J., Ekberg, K., Johansson, J., Henriksson, M., Pramanik, A., Johansson, B. I., Rigler, R., and Jörnvall, H. (2000) Role of C-peptide in human physiology. *Am. J. Physiol. Endocrinol. Metab.,* **278,** E759–E768.

Wahren, J., and Jörnvall, H. C. (2003) C-peptide makes a comeback. *Diabetes Metab. Res. Rev.,* **19,** 345–347.

Zierath, J. R., Handberg, A., Tally, M., and Wallberg-Henriksson, H. (1996) C-peptide stimulates glucose transport in isolated human skeletal muscle independent of insulin receptor and tyrosine kinase activation. *Diabetologia,* **39,** 306–313.

*Aaron Vinik*
*Norfolk, Virginia, February, 2004*

